# A model of hepatic steatosis with declined viability and function in a liver-organ-on-a-chip

**DOI:** 10.1038/s41598-023-44198-0

**Published:** 2023-10-09

**Authors:** Natsupa Wiriyakulsit, Ploychanok Keawsomnuk, Saowarose Thongin, Pimonrat Ketsawatsomkron, Kenjiro Muta

**Affiliations:** Chakri Naruebodindra Medical Institute, Faculty of Medicine Ramathibodi Hospital, Mahidol University, 111 Bang Pla, Bang Phli, Samut Prakan 10540 Thailand

**Keywords:** Lab-on-a-chip, Dyslipidaemias, Obesity, Experimental models of disease, Metabolic syndrome

## Abstract

Nonalcoholic fatty liver disease (NAFLD) begins with benign steatosis caused by ectopic storage of triacylglycerols in the liver. Persistent steatosis, in combination with other genetic and environmental factors, leads to nonalcoholic steatohepatitis (NASH) characterized by functional impairment, inflammation, and fibrosis. However, it remains unclear how persistent steatosis directly contributes to the progression of NAFLD, which may represent a therapeutic target. The organ-on-a-chip (OOC) has emerged as a new culture platform to recapitulate human pathological conditions under which drug candidates can be screened. Here, we developed a simple OOC steatosis model using the Mimetas OrganoPlate with a human liver cell line, HepG2. Treating the HepG2 OOCs with fatty acid overload induced steatosis within 24 h. Moreover, persistent steatosis for 6 days impaired OOC viability and hepatic function, as measured by a WST-8 assay and albumin production, respectively. Lastly, the HepG2 OOCs were exposed to drugs being tested in clinical trials for NAFLD/NASH during the 6-day period. Pioglitazone improved the OOC viability while elafibranor reduced the steatosis in association with reduced viability and albumin production. In conclusion, we show that the HepG2 steatosis OOC model is a useful tool on which the efficacy and toxicity of various therapeutic candidates can be tested.

## Introduction

Hepatic steatosis caused by the ectopic accumulation of triacylglycerols (TAGs) in the liver occurs in the early stage of nonalcoholic fatty liver disease (NAFLD), but the majority of patients present with a benign condition. Approximately 20–30% of NAFLD patients progress to nonalcoholic steatohepatitis (NASH)^1^, which eventually leads to cirrhosis, liver cancer, and death^2^. NASH is marked by cell damage and dysfunction resulting from inflamed residual immune cells, ballooning hepatocytes, and fibrosis^3^. Most NAFLD/NASH-directed studies focus on investigating the mechanism underlying the pathological development and seeking potential therapeutics. However, prior to and during NAFLD progression to the later stages, the influence of steatosis on hepatic viability and functions remains unclear.

The prevalence of NAFLD has increased in the past thirty years^4^. Current treatment options are largely dependent on lifestyle changes, as there are currently no efficacious therapeutic remedies for NAFLD approved by the US Food and Drug Administration (FDA) that can fully reverse NAFLD-induced hepatic damage^5^. Drug candidates specifically designed for NAFLD treatment are still under development due to patients’ differential responsiveness to them in clinical trials^6^. Moreover, given the causal relationship between NAFLD and type 2 diabetes (T2D)^7^, therapeutic potentials of drugs approved for T2D, such as metformin and pioglitazone, have been explored in clinical trials. These studies have demonstrated pioglitazone’s therapeutic capability to alleviate NAFLD despite its adverse effects on body weight^8^.

Ectopic accumulation of TAGs is believed to be an initial event that buffers an excess influx of non-esterified fatty acids (FAs) from dysregulated white adipose tissues (e.g., enhanced lipolysis under insulin-resistant conditions) and excessive intake of dietary fats into non-adipose tissues such as the liver^9^. Recent findings suggest that hepatocytic dysfunction and cell death may be triggered even before progression to NASH^10^. For example, reduced hepatic albumin production in patients with NAFLD has been proposed as a predictive marker of NASH-related events^11^. These lines of evidence led us to speculate that the process of steatosis-mediated liver deterioration could be a potential therapeutic target of NAFLD.

To study the mechanism underlying the pathological development of NAFLD, as well as to identify therapeutic candidates for this disorder, numerous in vivo and in vitro models of steatosis have been established to date^12,13^. Genetic modification and feeding with excessive amounts of nutrients are sufficient to develop steatosis in animal models of NAFLD. However, there are discrepancies in the genetic background and the reactions to drug exposure between human patients and animal models^12^. On the other hand, in two-dimensional (2D) culture models of NAFLD, fat accumulation can be induced by treating cells with excessive amounts of free FAs, high concentrations of glucose and insulin, or β-oxidation inhibitors^13^. However, these conventional 2D models allow us to examine whether investigational drugs are capable of decreasing fat accumulation within a short-term period that generally allows 24–48 h treatment with drugs, aside from the one study^14^. It is important to note that most NAFLD studies investigating preclinical drug candidates are designed to inhibit the process of fat accumulation in a short duration, but do not aim to reverse it^15^. This is a critical pitfall of the study design since long-term treatment with the drug candidates may be required for the normalization of fat accumulation. Therefore, the prevailing in vivo and in vitro testing platforms are largely incapable of recapitulating genetically corresponding and pathophysiologically-relevant conditions of NAFLD in humans^16^. Consequently, the paucity of human in vivo-like models may not only delay the product development process but also, more importantly, put the patients’ health unnecessarily at risk with unanticipated adverse effects of drug candidates^17^.

Organ on-a-chip (OOC) technology has emerged as a next-generation platform for drug discovery since recent methodological advances have enabled us to culture human cells in a 3D cellular architecture designed to faithfully mimic the microenvironment and functionality of the organs^18^. Multiple OOC models of NAFLD and NASH have been established on commercially available or academically invented chips^13,19–21^. These models have effectively captured NAFLD and NASH pathological features, such as accumulated fat contents and responsiveness to long-term drug treatment. However, they lack high-throughput capacity. This is likely due to convoluted designs in chip structure and the inclusion of multiple non-parenchymal cells, such as Kupffer cells, liver sinusoidal endothelial cells, and hepatic stellate cells^19,22^. Therefore, these sophisticated OOC models of NAFLD/NASH represent human pathophysiologically-relevant platforms that can be used to study the mechanisms underlying disease progression but remain inefficient for large-scale screening of chemical compounds. Rather, a simple model that features some key clinical outcomes of NAFLD in a high-throughput OOC platform could be employed in the preliminary screening of compound libraries^23^.

Here, we developed a simple OOC model of steatosis on the Mimetas OrganoPlate with high-throughput capacity using HepG2, a human liver cell line. This model successfully recapitulated NAFLD-related pathologies, in which short exposure to excess amounts of FAs caused non-toxic steatosis, while persistent steatosis led to hepatocytic and functional deteriorations in 6 days. Further, we tested investigational medications that have been clinically evaluated for human trials on the HepG2 steatosis OOC model in the 6-day period.

## Results

### Characterization of a liver OOC model with collagen extracellular matrix (ECM)

**Figure 1:**
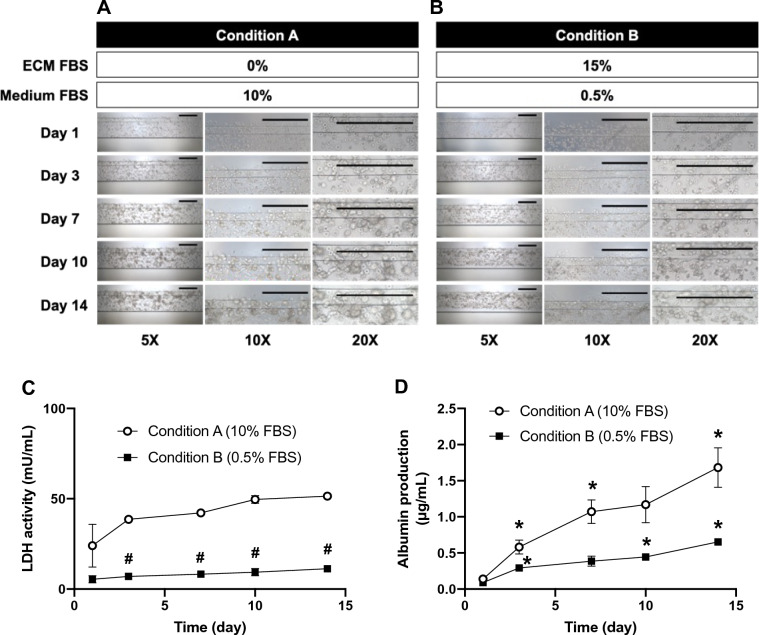
Characterization of a liver OOC model with collagen ECM. HepG2 OOC cultures were maintained under two different conditions (**A** and **B**). HepG2 cells were embedded in collagen ECM containing no FBS (**A**) or 15% FBS (**B**) with William’s E medium including 10% FBS (**A**) or 0.5% FBS (**B**), respectively. Images of HepG2 OOCs were taken on Days 1, 3, 7, 10, and 14 at 5X, 10X, and 20X magnifications. Scale bar = 500 μm. Conditioned media were collected on Days 1, 3, 7, 10, and 14 to quantify LDH activity (**C**) and albumin concentrations (**D**) in HepG2 OOCs under Conditions A or B. Data are presented as mean ± SEM and analyzed by a two-way repeated measures ANOVA (n = 4–6). *p < 0.05 versus Day 1 of each condition. #*p* < 0.05 versus Condition A.

The Mimetas OrganoPlate has previously been used to establish a liver OOC model with spherical HepG2 cells embedded in Matrigel ECM, which provides a three-dimensional (3D) scaffold and critical growth factors^24,25^. Accordingly, we first attempted to replicate the liver OOC preliminary to developing an OOC model of hepatic steatosis. As previously reported^24^, HepG2 OOCs grown in Matrigel ECM showed a greater production of albumin (which represent a functional capability of the liver) than HepG2 2D cultures (Supplementary Fig. 1A). Moreover, unlike long-term 2D HepG2 cultures, which had reduced cell viability over time^26^ (Supplementary Fig. 1B), the HepG2 OOCs retained stable viability for 2 weeks, as indicated by the lactate dehydrogenase (LDH) activity assay (Supplementary Fig. 1B). However, after Day 7, we observed HepG2 cells had migrated away from the Matrigel ECM into medium paths, where the cells formed 2D-like structure (data not shown) and likely received direct contact with a luminal flow of medium. By Day 14, the medium paths were thoroughly occupied by the 2D-like HepG2 cells (Supplementary Fig. 1C). These findings led us to conclude that the motility of HepG2 cells cultured in Matrigel ECM was inadequate to recapitulate physiologically-relevant characteristics of hepatocytes, especially when cultured for longer than 7 days. Therefore, we then decided to seek alternatives to Matrigel ECM that were suitable for the HepG2 OOC model of steatosis. Given that collagen-based ECM has been utilized to create 3D scaffolds for a number of various OOC models^27,28^, we selected collagen-I for the following OOC studies. Moreover, to compare the effects of fetal bovine serum (FBS)-derived nutritional and growth supplements on the characteristics of HepG2 OOC cultures, we designed the following two culture conditions (Fig. 1A and B): A) No FBS in collagen ECM with William’s E medium containing 10% FBS (Condition A that is comparable to conventional 2D culture rich in lipids and growth factors); B) 15% FBS in collagen ECM with William’s E medium containing 0.5% FBS (Condition B that mimics a poor energy state including limited amounts of lipid and growth factors). Under Conditions A and B, cell density and morphological changes of HepG2 cells in collagen ECM over a period of 14-day culture were consistent and reproducible (Fig. 1A and B). HepG2 cells under Condition A formed clusters at early time points, eventually turning into aggregated spheroids (Fig. 1A) in a manner similar to those grown in Matrigel ECM^29^ (Supplementary Fig. 1C). Conversely, Condition B induced less cell aggregation than Condition A (Fig. 1B). Next, the viability of the HepG2 OOC cultures was assessed by measuring LDH activity in conditioned medium collected on Days 1, 3, 7, 10, and 14. The LDH activity of HepG2 cells cultured under Condition B was maintained lower than that of HepG2 cells under Condition A over a period of 14 days, except for on Day 1 (Fig. 1C). From Day 3 to Day 14, the LDH activity under Condition A remained unchanged. This is similar to that of HepG2 OOCs with Matrigel ECM (Supplementary Fig. 1B). These viability results indicated that HepG2 OOCs with collagen ECM were highly viable for at least 2 weeks regardless of the variability of energy and growth factors. Albumin production is a defining function of the liver that is recapitulated in several liver OOC models^24,30^. To confirm this functional feature in our model, we measured albumin concentrations in conditioned media collected on Days 1, 3, 7, 10, and 14. The albumin-producing activity of HepG2 OOC cultures under Condition A increased incrementally over the experimental period in the same manner as that of HepG2 OOCs with Matrigel (Fig. 1D and Supplemental data Fig. 1A), while the HepG2 OOCs under Condition B produced albumin to a lesser extent (Fig. 1D). Collectively, these results demonstrate that the HepG2 OOC cultures with collagen can retain the physiological characteristics of hepatocytes more appropriately than conventional 2D cultures in a similar manner to the Matrigel ECM-developed HepG2 OOC cultures.

### Induction of hepatic steatosis by exposure to FAs

**Figure 2:**
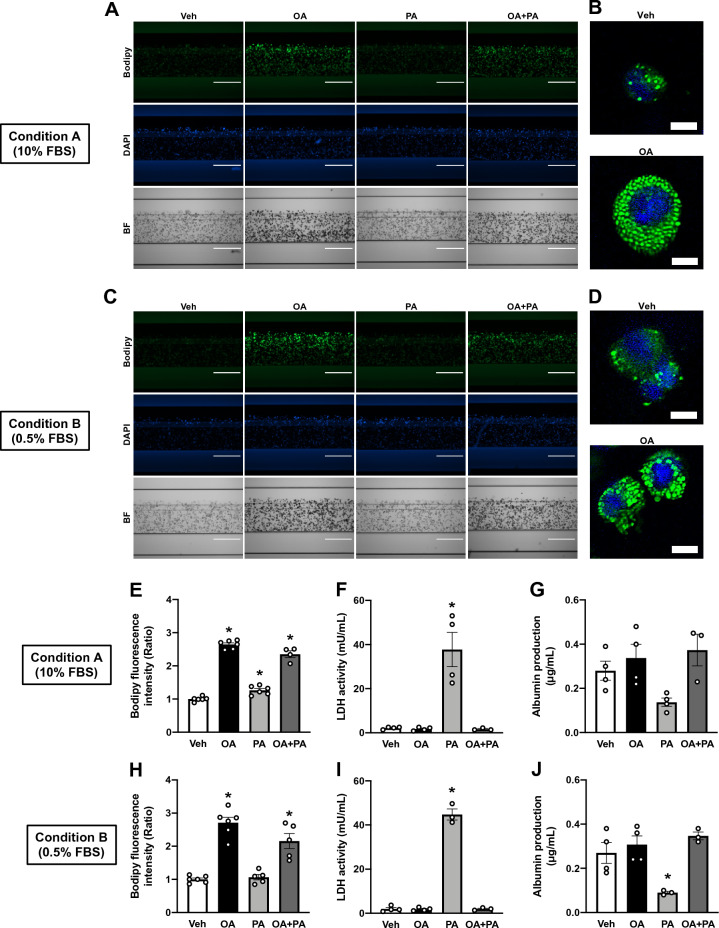
Induction of hepatic steatosis by exposure to fatty acids. HepG2 OOC cells were treated with vehicle (Veh, 1 mM NaOH), oleic acid (OA, 0.5 mM), palmitic acid (PA, 0.5 mM), or a mixture of OA and PA (OA + PA, 0.5 mM in total) for 24 h under Conditions A or B. (**A**–**D**) Representative fluorescence (**A** and **C**) or confocal (**B** and **D**) images of HepG2 OOC cells stained with Bodipy (green) or DAPI (blue). The confocal images were captured and processed using Zeiss ZEN 3.4 (blue edition) software. Bright-field (BF) images are shown in gray scale. (**E** and **H**) Quantitative analysis of Bodipy fluorescence in HepG2 OOCs. (**F** and **I**) LDH activity in conditioned medium of HepG2 OOC cultures. (**G** and **J**) Albumin concentration in conditioned medium of HepG2 OOC cultures. Data are presented as mean ± SEM and analyzed by one-way ANOVA (n = 3–6). *p < 0.05 versus Veh.

Exposing hepatocytes to excessive amounts of FA(s) to induce steatosis has been employed in numerous studies irrespective of cell culture system type (i.e., 2D, organoid, and OOC)^13^. Particularly, HepG2 2D cultures have been intensively studied^13^ and shown to mostly express key regulators of hepatic lipid metabolism^31^. However, as the gene expression profile of HepG2 OOCs may differ from that of the 2D cultures^32^, we compared the mRNA expression levels of 9 regulators involved in various lipid metabolic processes between 2D and OOC cultures of HepG2: Fatty acid binding protein 1 (FABP1) for fatty acid uptake; Apolipoprotein B (APOB) for very low-density lipoprotein secretion; Acetyl CoA carboxylase alpha (ACACA) for de novo lipogenesis; Diacylglycerol O-acetyltransferase 2 (DGAT2) for esterification; Patatin like phospholipase domain containing 2 (PNPLA2) for lipolysis; Carnitine palmitoyltransferase 1A (CPT1A) for beta-oxidation; Peroxisome proliferator-activated receptors alpha (PPARA), delta (PPARD) and gamma (PPARG) for transcription factors. The mRNA levels of FABP1, APOB, DGAT2, and PPARs were comparable between the two culture systems of HepG2 (Supplementary Fig. 2A,B,D, and G–I, respectively). In contrast, culturing HepG2 cells in the OOC units resulted in reduced expression of ACACA (Supplementary Fig. 2C) and increased expression of PNPLA2 and CPT1A (Supplementary Fig. 2E-F) relative to those of 2D HepG2 cultures. These changes in the HepG2 transcripts were consistent with our functional studies that assessed the capabilities of de novo lipogenesis and lipolysis in HepG2 OOCs (Supplementary Fig. 3A and B). These data demonstrated the mechanistic basis for the development of a steatosis model using HepG2 OOCs by exposure to FAs, but not by activation of de novo lipogenesis. To establish a steatosis model of HepG2 OOC, we incubated the cells with vehicle control (Veh, 1 mM NaOH), oleic acid (OA, 0.5 mM), palmitic acid (PA, 0.5 mM), or a mixture of OA and PA (OA + PA, 0.5 mM in total) for 24 h under Conditions A or B. Neutral lipids and nuclei in HepG2 OOCs were stained with Bodipy (green) and DAPI (blue) dyes, respectively. The fluorescence signals were visualized (Fig. 2A and C) and quantified (Fig. 2E and H) using a cell-imaging plate reader. To confirm the lipid droplet formation, the confocal microscopy images were taken in Veh- or OA-treated HepG2 OOCs (Fig. 2B and D). OA treatment apparently increased the number of lipid droplets in HepG2 OOCs under both Conditions A and B. Relative to the Veh control, OA treatment caused an approximately 2.5-fold increase in the fluorescence intensities of Bodipy (Fig. 2A–E and H). In contrast, 24 h exposure to PA slightly but significantly induced fat accumulation under Condition A (Fig. 2E) but was insufficient under Condition B (Fig. 2H). This is dissimilar to PA-evoked steatosis in HepG2 2D cultures in our and others’ studies (Supplementary Fig. 4)^33–35^. OA + PA treatment produced an intermediate accumulation of lipid droplets (Fig. 2E and H). Next, we evaluated whether FA-induced lipid accumulation influenced the viability and functional activity of HepG2 OOCs. The LDH assay revealed significant reductions in OOC viability in PA-exposed cells alone (Fig. 2F and I), which coincided with a trending decrease in albumin production under Condition A (Fig. 2G) and a significant decrease in production under Condition B (Fig. 2J). On the other hand, treating HepG2 OOCs with OA or OA + PA for 24 h made no impact on OOC viability and albumin production (Fig. 2F,G and I,J). Collectively, these data suggested the feasibility of using HepG2 OOCs exposed to OA or a mixture of OA and PA as a model for steatosis.

### Effects of persistent steatosis on OOC viability and albumin production in HepG2 steatosis OOCs

**Figure 3:**
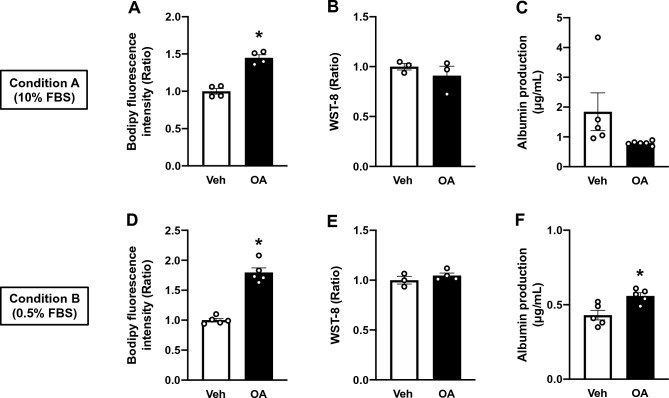
Effects of 6-day persistent steatosis on cell viability and albumin production in HepG2 OOCs exposed to OA for 24 h. HepG2 OOC cells were treated with vehicle (Veh, 1 mM NaOH) or oleic acid (OA, 0.5 mM) for 24 h to induce steatosis and then maintained in complete media for Conditions A or B for 6 days. (**A** and **D**) Quantitative analysis of Bodipy fluorescence. (**B** and **E**) WST-8-based viability assay. (**C** and **F**) Albumin concentration in conditioned medium. Data are presented as mean ± SEM and analyzed by Student’s t-test (n = 3–6). *p < 0.05 versus Veh.

As ectopic deposition of TAGs in the liver has been suggested as a buffering mechanism that sequesters toxic accumulation of non-esterified FAs^36,37^, hepatic steatosis is mostly asymptomatic, especially at the onset of NAFLD^2^. Nearly 20–30% of NAFLD patients develop NASH with inflammatory circumstances that induce ballooning and apoptosis to hepatocytes^1,2^. However, whether the other 70–80% of patients maintain benign conditions or present with hepatic impairment to some extent over time remains unclear. Here, we explored the influence of persistent steatosis on OOC viability and function of hepatocytes in steatosis-induced HepG2 OOCs. To this end, following 24 h steatosis induction with OA, HepG2 OOC cultures were maintained under Conditions A or B for 6 days (without additional FA treatments). OA-induced fat accumulation was retained in the HepG2 OOCs under both Conditions; however, the fold changes in the Bodipy fluorescence between Veh and OA treatments (Fig. 3A and D) appeared to be less intense than those observed 24 h after steatosis induction (Fig. 2E and H). Moreover, the viability of OA-treated HepG2 OOC, as assessed by a WST-8 assay, was unchanged after 6 days in culture relative to that of the Veh-treated group (Fig. 3B and E). Consistently, persistent steatosis caused no significant reductions in albumin production in HepG2 OOCs exposed to OA as compared to those exposed to Veh (Fig. 3C and F). These data demonstrate that persistent steatosis caused no detrimental effects on the hepatocytes 6 days after fat accumulation induced by OA, suggesting that OA-induced steatosis in HepG2 OOCs was likely to replicate the benign condition of NAFLD.

**Figure 4:**
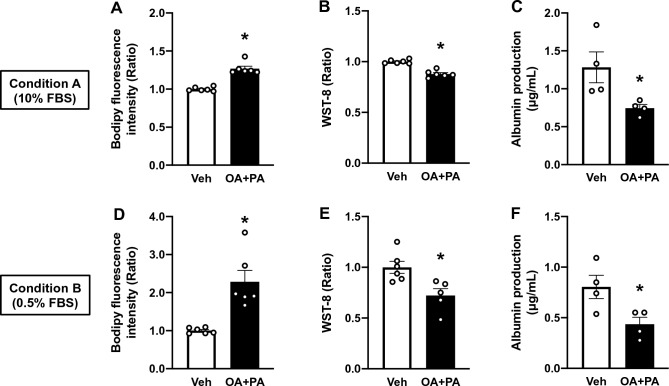
Effects of 6-day persistent steatosis on cell viability and albumin production in HepG2 OOCs exposed to OA + PA for 24 h. HepG2 OOC cells were treated with vehicle (Veh, 1 mM NaOH) or a mixture of oleic acid and palmitic acid (OA + PA, 0.5 mM in total) for 24 h to induce steatosis and then maintained in complete media for Conditions A or B for 6 days. (**A** and **D**) Quantitative analysis of Bodipy fluorescence. (**B** and **E**) WST-8-based viability assay. (**C** and **F**) Albumin concentration in conditioned medium. Data are presented as mean ± SEM and analyzed by Student’s t-test (n = 4–6). *p < 0.05 versus Veh.

OA has been reported to be more steatosis-inducing than PA, while PA is more cytotoxic than OA in hepatocyte 2D cultures^38^. In humans, both OA and PA are major constituents of plasma TAGs, such that exposing hepatocytes to an excess amount of a mixture of OA and PA would more effectively represent the lipid profile observed in patients with NAFLD/NASH^13^. Therefore, we next exposed HepG2 OOCs to a mixture of OA and PA at a total concentration of 0.5 mM for 24 h, followed by 6-day culturing without additional FAs. The state of steatosis was preserved in HepG2 OOCs under the both Conditions (Fig. 4A and D). The WST-8 assay revealed a reduction in the viability of HepG2 OOCs treated with OA + PA relative to one treated with Veh (Fig. 4B and E). Along with these reductions in viability, albumin production was decreased by ~ 40% in HepG2 OOCs exposed to the FA mixture (Fig. 4C and F). This finding is in accordance with a previous report that showed a decline in albumin concentrations in the serum of patients with NAFLD precedes NASH-associated adverse events^11^. These data suggest that HepG2 OOCs with steatosis induced by OA + PA might recapitulate the pathological changes in NAFLD patients who eventually experience severe liver-related deficits involved in NASH^11^.

### Effects of 6-day treatment with metformin, pioglitazone, or elafibranor on fat accumulation, viability, and albumin production in HepG2 steatosis OOCs

**Figure 5:**
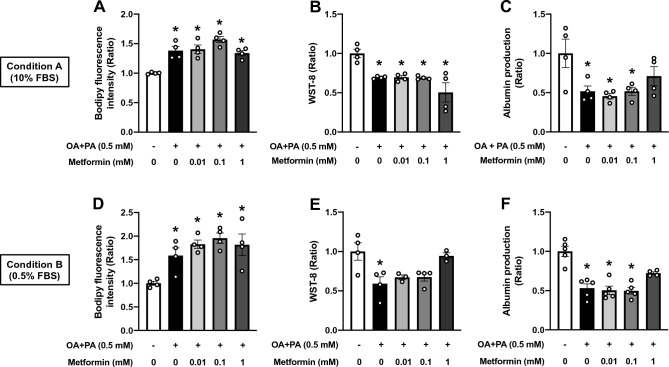
Effects of metformin on fat accumulation, cell viability, and albumin production in HepG2 steatosis OOCs. HepG2 OOC cells were treated with vehicle (Veh, 1 mM NaOH), a mixture of oleic acid and palmitic acid (OA + PA, 0.5 mM in total) for 24 h to induce steatosis and then cultured in 10% or 0.5% FBS-containing media (Conditions A or B, respectively) with vehicle (serum-free medium) or metformin (0.01, 0.1, or 1 mM) for 6 days. (**A** and **D**) Quantitative analysis of Bodipy fluorescence. (**B** and **E**) WST-8-based viability assay. (**C** and **F**) Albumin amount (ratio) in conditioned medium. Data are presented as mean ± SEM and analyzed by one-way ANOVA (n = 3–5).

There are several drugs for T2D approved by the US FDA, such as metformin and pioglitazone. The efficacy and safety of these T2D drugs have been tested in patients with NAFLD in clinical trials^8^. Metformin yielded no significant improvements to steatosis and NAFLD activity score (NAS)^8^, even though favorable results were shown in preclinical studies^15,39^. On the other hand, pioglitazone reduced steatosis, NAS, and hepatic enzyme activity but showed safety concerns over weight gain^8^. In addition, an experimental compound, elafibranor (a PPARα/δ dual agonist) specifically designed for cardiometabolic diseases, has been tested in clinical trials in patients with NAFLD/NASH. Elafibranor exhibited hepatoprotective properties by reducing hepatic fat storage and enzyme activity^40^. Here, we attempted to reproduce these clinical trial outcomes using the HepG2 steatosis OOC system. The effectiveness of these drug candidates has been supported by previous in vitro and in vivo animal studies^15,39,41^. However, these preclinical studies were largely designed to expose to the drug during the processes of steatosis induction (in vitro) except for a few studies ^14,23^ or NAFLD/NASH development (in vivo). In fact, in patients, drug treatment was initiated following the diagnosis of steatosis. To this end, we treated HepG2 OOCs with these medications for 6 days following the induction of steatosis by 24 h exposure to OA + PA. Similar to metformin clinical trials that showed no improvement in hepatic steatosis^8^, 6-day treatment with various concentrations of metformin (0.01, 0.1, or 1 mM) exerted no fat-reducing effect in HepG2 steatosis OOCs under both Conditions A and B (Fig. 5A and D). Similarly, metformin did not affect OOC viability and albumin production (Fig. 5B,C and E,F), both of which were reduced by the 6-day persistent steatosis (Fig. 4B,C and E,F). We concluded that unlike previous 2D in vitro and animal models of NAFLD^15,39^, 6-day metformin treatment was unable to reverse persistent steatosis-associated hepatic deteriorations in HepG2 OOCs.

**Figure 6:**
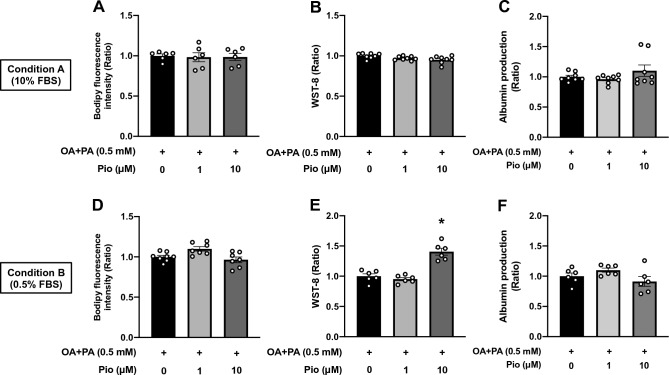
Effects of pioglitazone on fat accumulation, cell viability, and albumin production in HepG2 steatosis OOCs. HepG2 OOC cells were treated with a mixture of oleic acid and palmitic acid (OA + PA, 0.5 mM in total) for 24 h to induce steatosis and then cultured in 10% or 0.5% FBS-containing media (Conditions A or B, respectively) with vehicle (0.1% DMSO) or pioglitazone (Pio, 1 or 10 μM) for 6 days. (**A** and **D**) Quantitative analysis of Bodipy fluorescence. (**B** and **E**) WST-8-based viability assay. (**C** and **F**) Albumin amount (ratio) in conditioned medium. Data are presented as mean ± SEM and analyzed by one-way ANOVA (n = 6–8). *p < 0.05 versus OA + PA in the absence of pioglitazone.

Next, using the same treatment protocol as metformin, pioglitazone was tested on HepG2 steatosis OOCs. In disagreement with clinical trial data that found that pioglitazone treatment ameliorated liver fat^8^, neither 1 nor 10 μM of pioglitazone normalized fat accumulation induced by 24 h exposure to OA + PA (Fig. 6A and D). This is probably because in previous human studies, pioglitazone reduced FAs influx into the liver by enhancing lipid anabolism in adipose tissues^42,43^. We found no benefit of pioglitazone to OOC viability and albumin production under Condition A (Fig. 6B and C), while 10 μM pioglitazone yielded significant benefits to viability without affecting albumin release under Condition B (Fig. 6E and F). These results suggest that pioglitazone was effective in eliciting hepatoprotective effects in an energy-status-dependent manner, while this T2D medication had no apparent impact directly on the lipid metabolism of hepatocytes.

**Figure 7:**
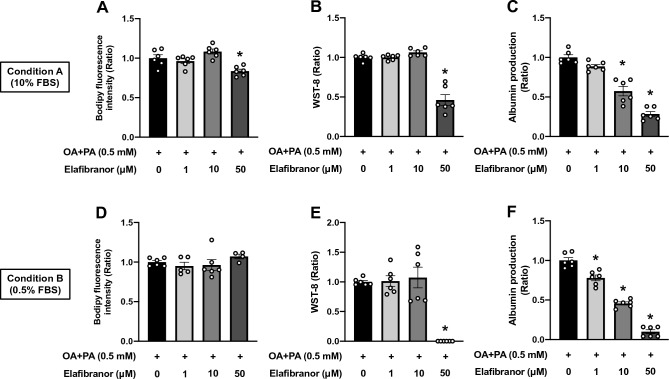
Effects of elafibranor on fat accumulation, cell viability, and albumin production in HepG2 steatosis OOCs. HepG2 OOC cells were treated with a mixture of oleic acid and palmitic acid (OA + PA, 0.5 mM in total) for 24 h to induce steatosis and then cultured in 10% or 0.5% FBS-containing media (Conditions A or B, respectively) with vehicle (0.1% DMSO) or elafibranor (1, 10 or 50 μM) for 6 days. (**A** and **D**) Quantitative analysis of Bodipy fluorescence. (**B** and **E**) WST-8-based viability assay. (**C** and **F**) Albumin amount (ratio) in conditioned medium. Data are presented as mean ± SEM and analyzed by one-way ANOVA (n = 4–6). *p < 0.05 versus OA + PA in the absence of elafibranor.

Lastly, HepG2 steatosis OOCs were treated with elafibranor for 6 days after a 24 h induction of steatosis. The fluorescence intensity of Bodipy was significantly decreased by elafibranor at a concentration of 50 μM but not at 1 and 10 μM under Condition A (Fig. 7A). This fat-reducing effect was not evident in that under Condition B (Fig. 7D). Persistent steatosis-mediated reductions in OOC viability and albumin production were deteriorated by elafibranor (Fig. 7B,C and E,F). In accordance with the clinical trial^40^, elafibranor produced an effect against ectopic fat accumulation in HepG2 steatosis OOC, but this effect seemed to be associated with reductions in cell viability and albumin production.

## Discussion

In the current study, we developed HepG2 steatosis OOCs with hepatocytic and functional deteriorations. Our study demonstrated that 1) steatosis can be induced by treating HepG2 OOCs with OA or a mixture of OA and PA for 24 h, 2) OOC viability and albumin production were significantly reduced 6 days after the establishment of steatosis in response to 24 h treatment with OA + PA but not with OA alone, 3) some of the hepatic responses to drug candidates for NAFLD/NASH in humans were recapitulated in HepG2 steatosis OOCs. These findings suggest that HepG2 steatosis OOCs can be employed to screen drug candidates that are efficacious in normalizing fat accumulation and viability of hepatocytes. In addition, this model may be useful for identifying the potential toxicities of therapeutic candidates.

Various OOC models of NAFLD/NASH have been developed by both academic labs and pharmaceutical/biotech companies using custom or commercially available OOCs^13,19–21^. Most of the studies that utilized parenchymal (i.e., hepatocytes derived from cell lines, primary human cells, and inducible pluripotent stem cells) and non-parenchymal cells (i.e., sinusoidal endothelial cells, Kupffer cells, and hepatic stellate cells, derived from cell lines or primary cells) claimed that the models were able to successfully recapitulate the pathological characteristics of NAFLD/NASH^13^. Moreover, because OOC systems allow for long-term culture, the development of NAFLD/NASH in OOC models can be prevented by chronic treatment with investigational drugs during which FA overload induces steatosis^19,20^. However, the limitation of these sophisticated models is the workload capacity to screen large-scale chemical libraries. To compensate for this restriction, we tested the hypothesis that a simple OOC model of steatosis with a hepatic cell line might be sufficient to replicate some of the biological features of the liver, the pathology of NAFLD, and the drug responses found in humans, but with some limitations such as inflammatory responses generally mediated by the non-parenchymal cells^44^.

Morphological characteristics and cellular functions of hepatocytes are altered by static 2D culture platforms^45^. Conversely, hepatocytes in 3D OOC culture systems with a flow of cell culture medium have been reported to maintain physiological characteristics and sustenance with a steady supply of nutrients and oxygen and removal of cellular wastes^46,47^. Therefore, it is critical to establish NAFLD OOCs that possess pathophysiologically-relevant characteristics and functions for drug-efficacy testing. In the current study, we first attempted to replicate the biological features of the liver with collagen-embedded HepG2 cells in the Mimetas OrganoPlate that has high-throughput capacity. Considering several advantages and disadvantages in the selection of cell types is an important key step for successful OOC development. For this report, the HepG2 cell line was selected due to its accessibility, assay reproducibility, and previous data in 2D culture systems for comparison, all of which are advantages in establishing a high-throughput screening. Moreover, HepG2 cells have a PNPLA3 I148M mutation that was identified as a factor of genetic susceptibility in NAFLD patients^48,49^. Even though HepG2 cells originated from human hepatocellular carcinoma, the sizes of HepG2 cluster areas in OOCs were unchanged over 2 weeks of experimental periods^24^, suggesting suppression of HepG2 proliferation in OOC platforms. Blood concentrations of nutrients and growth factors greatly fluctuate depending on the circadian rhythm and the timing of energy consumption in animals^50^. However, particularly in NAFLD/NASH OOC models, cell cultures have been maintained in a serum-free medium to avoid confounding effects of serum components such as nutrients, growth factors, and serum protein on disease progression and drug binding^19,20^. To fairly consider these possibilities, we utilized media containing two different concentrations of FBS. The Condition A medium with 10% FBS consisted of a greater amount of lipids, growth factors, and serum proteins that may sequester drugs, while the medium with 0.5% FBS for Condition B provided HepG2 OOCs with limited amounts of these serum components. Comparable to other OOC models of the liver^24^, HepG2 OOC under Condition A enhanced albumin production to a greater extent than Condition B, and the overall viabilities of HepG2 OOCs under Conditions A and B were stable in the experimental period of 2 weeks. Interestingly, our data indicated that serum-derived factors seemingly affected OOC viability and functional assessments when HepG2 steatosis OOCs were treated with drugs. Collectively, in agreement with other OOC models of the liver and NAFLD, HepG2 OOCs with collagen ECM were likely to maintain structural stability, viability, and functional integrity for at least 2 weeks in a more physiologically-relevant manner than conventional 2D cultures of hepatocytes^24^.

Induction of steatosis in hepatocytes by exposure to FAs is the gold-standard method in 2D, organoid, and OOC models of NAFLD. The mixture of OA and PA has been typically used to mimic the lipid profile in the bloodstream of patients with NAFLD or obesity^13^. In most 2D models of steatosis, either of OA, PA, or OA + PA at concentrations ranging from 100 μM to 1 mM are able to induce steatosis within 24 h. Similarly, the OA + PA approach is sufficient for steatosis induction in OOC models^19,20,23,51^. In line with previous 2D and OOC models of NAFLD using HepG2 cells, steatosis was successfully induced in our HepG2 OOC model in response to OA or a mixture of OA and PA; whereas, PA failed or was insufficient to induce steatosis. HepG2 OOCs are likely less sensitive to PA exposure for inducing TAG synthesis than 2D HepG2 cultures in which PA increases TAG accumulation^33–35^. On the other hand, PA-induced hepatocellular toxicity was evident. This finding suggests an advantage of HepG2 OOCs over 2D culture systems in terms of studying PA-dependent pathological outcomes without being affected by lipid accumulation. Intriguingly, in the concomitant presence of OA (an unsaturated FA), PA failed to elicit deleterious effects on OOC viability and albumin production, supporting the idea that TAG synthesis promoted by OA offers a buffering mechanism against exposure to cytotoxic PA^36,37^. In 2D HepG2 cultures, OA-induced steatosis has been shown to cause reduced cell proliferation in association with apoptosis^52^. In contrast, 24 h exposure to OA, followed by maintaining persistent steatosis for 6 days had no negative impact on the viability and albumin production in HepG2 OOCs. This is consistent with the report that unsaturated FAs such as OA inhibit oxidative stress and inflammatory responses in patients with NASH^53^. Moreover, liver levels of OA are reciprocally correlated with the occurrence of fibrosis partly resulting from chronic liver damage^54^. Thus, OA-induced steatosis in HepG2 OOCs seems to represent an asymptomatic condition of NAFLD. Conversely, 6-day persistent steatosis initially induced by 24 h treatment with OA + PA caused reductions in the viability and albumin production in HepG2 steatosis OOCs. The reduced viability indicated a possible pathogenic role of hepatocytes in NAFLD progression in response to persistent steatosis without receiving inflammatory inputs from nonparenchymal cells^3^. Moreover, the albumin production decreased by persistent steatosis may correlate with the fact that NAFLD patients with severer clinical outcomes have reduced serum concentrations of albumin^11^.

The development of drug screening platforms largely relies on how the disease models recapitulate the pathological condition and the responsiveness to drug candidates in humans. The limitation of HepG2 steatosis OOCs is the lack of inflammatory responses that require non-parenchymal cells in the liver, which are major contributors to the progress to NASH^3^. However, several lines of evidence support the idea that NAFLD progression to NASH may be partly attributed to damaged hepatocytes independent of inflammatory circumstances^10^. Therefore, we examined whether three major drug candidates for NAFLD/NASH alleviated fat accumulation and reductions in viability and albumin production in HepG2 steatosis OOCs. Metformin is an insulin sensitizer for the treatment of T2D and has been suggested to ameliorate fat accumulation and inflammation via various mechanisms in preclinical settings^15,39^. However, recent clinical studies that evaluated the efficacy of metformin showed no significant benefit to patients with NAFLD/NASH^8^. Consistently, 6-day treatment with metformin exerted no beneficial effects on fat accumulation, viability, and albumin production in HepG2 steatosis OOCs. In contrast, pioglitazone, a peroxisome proliferator-activated receptor gamma (PPARγ) agonist that is also used as a medication for T2D, has been shown to be effective in decreasing steatosis, NAS, and liver damage^8^. This was partially replicated in HepG2 steatosis OOCs treated with 10 μM pioglitazone under Condition B, which showed improved viability. However, pioglitazone failed to counteract steatosis in our model, which is inconsistent with results from human clinical trials^8^. This discrepancy may be explained by the previous tissue-specific PPARγ knockout studies in mice^43^. NAFLD models of mice rendered deficient in hepatocyte-specific PPARγ had reduced hepatic steatosis relative to PPARγ-intact counterparts, whereas adipose tissue-specific PPARγ knockout mice were lipodystrophy but associated with fatty liver. Therefore, under physiological circumstances, pioglitazone seems to reduce FAs influx into the liver by enhancing lipogenesis and suppressing lipolysis in fat tissues. In our model, we found that pioglitazone had no direct impact on hepatic lipid catabolism. Elafibranor, a dual agonist for PPARα/δ, has been tested in several clinical trials, demonstrating hepatoprotective properties by reducing hepatic fat storage and hepatic enzyme activity in the blood^40^. In a similar manner to that reported in humans, exposing HepG2 steatosis OOCs to elafibranor for 6 days significantly reduced fat accumulation. However, this anti-steatosis activity seemed to be specifically associated with adverse effects of elafibranor at 50 μM, as demonstrated by the reductions in cell viability and albumin production. As yet, to the best of our knowledge, no indication of elafibranor toxicity was identified in clinical trials^55^. Our data suggest possible detrimental effects of elafibranor in patients, which should be considered for the determination of optimal doses in future clinical trials. Moreover, we show the promise of utilizing HepG2 steatotic OOCs for drug toxicity study.

In conclusion, we successfully developed a steatosis OOC model with declined cell viability and function in a commercially-available microfluidic device designed for high-throughput screening (Mimetas OrganoPlate). HepG2 steatosis OOCs offer a drug efficacy and toxicity testing platform to assess whether compounds of interest can improve or exacerbate steatosis, cell damage, and albumin production. Due to its high screening capacity, low cost, and reduced workload, this platform may be useful for the preliminary screening of drug candidates for treatment of NAFLD/NASH.

## Methods

### Chemical preparation

Oleic acid (OA, Sigma-Aldrich, US) and palmitic acid (PA, Sigma-Aldrich, US) were dissolved in 0.1N NaOH (Sigma-Aldrich, US) by heating at 90 °C on a heat block. A preparation of 10% fatty acid-free bovine serum albumin (BSA, Sigma-Aldrich, US) was made in serum-free minimum essential media (MEM, Gibco, US). Metformin (Sigma-Aldrich, US) was diluted in serum-free MEM to make 1 M stocks. Pioglitazone (Tocris, US) and elafibranor (MedChemExpress, US) were dissolved in 100% anhydrous dimethylsulfoxide (DMSO, Sigma-Aldrich, US) to make 10 mM and 50 mM stocks, respectively. Prepared chemicals were aliquoted and stored at  − 20 °C.

### HepG2 2D cultures

HepG2 cells (human hepatocarcinoma) were purchased from ATCC (HB-8065) and were grown in Dulbecco’s Modified Eagle’s Medium (DMEM, Gibco, US) supplemented with 10% FBS (Sigma-Aldrich, US), 1 mM sodium pyruvate (Gibco, US) and 2 mM GlutaMAX (Gibco, US) at 37 °C in a humidified incubator with 95% air and 5% CO_2_. When the confluency reached 70–80% in 100 mm dishes, HepG2 cells were subjected to the following experiments. For comparison with HepG2 OOC cultures, HepG2 2D cultures were prepared in William’s E medium (11 mM D-glucose, Gibco, US) containing FBS (10%, Hyclone, US) and 2 mM L-glutamine (Gibco, US) in 96-well plates at a concentration of 2 × 10^4^ cells/well. The conditioned medium (~ 100 μL) in which the 2D cells had been incubated for 24 h was collected on Days 1, 3, 7, and 14 for experiments.

### HepG2 OOC cultures

To culture HepG2 cells in OrganoPlate 2-lane plates (cat#9605-400-B, Mimetas, Netherlands), we followed an OOC protocol for hepatocytes that had been established elsewhere^24,25^. HepG2 cells were suspended in Matrigel (Corning, US) or Cultrex Collagen I (4 mg/mL, rat tail, Bio-Techne, US) at a density of 1 × 10^7^ cells/mL with 15% FBS or none. The cell suspension (~ 3.0 μL) was infused into a gel inlet chamber of the OOC. HepG2 OOC cultures were replenished daily with 100 μL of William’s E medium containing FBS (0.5 or 10%) and 2 mM L-glutamine. The plate was maintained on an interval rocker (switching sides every 7 min at 8°) that creates a flow of medium in a CO_2_ incubator at 37 °C according to the manufacturer’s instruction. On Days 1, 3, 7, 10, and 14, the morphologies of HepG2 OOC cultures were recorded and the conditioned medium with which the 3D cells had been incubated for 24 h was collected and stored at  − 80 °C until use.

### Induction of steatosis in HepG2 OOCs with fatty acids

At 24 h after seeding, HepG2 OOCs were treated with Veh (1 mM NaOH), 0.5 mM oleic acid (OA), 0.5 mM palmitic acid (PA), or a mixture of OA and PA (1:1 ratio = 0.25 mM: 0.25 mM, respectively) in the complete William’s E medium containing 1% fatty acid-free BSA that is the carrier of FAs for 24 h. Fat accumulation was confirmed by observing lipid droplets in the FA-treated groups under a microscope.

### WST-8 assay for determination of viability

A WST-8 assay was used to quantify the viability of HepG2 OOC cultures in some experiments. Following medium removal, 25 μL of the WST-8 solution (Abcam, UK) was added to each medium chamber. The plates were incubated for 2 h on the interval rocker in the CO_2_ incubator at 37 °C. The absorbance of WST-8 signals was measured at 450 nm with a plate reader.

### Lactate dehydrogenase (LDH) assay

To assess cell viability in some experiments, LDH activity in the conditioned medium was quantified by an LDH Activity Assay Kit (Sigma-Aldrich, US). The optical density at 450 nm was measured every 5 min after initiating the assay reaction to calculate the LDH activity.

### Staining neutral lipids of HepG2 OOCs with Bodipy

Medium ports were washed with phosphate-buffered saline (PBS) for 6 min, 5 times on the interval rocker (switching sides every 2 min at 5°), and then fixed with 4% paraformaldehyde for 30 min. 5 µM Bodipy (Thermo Fisher Scientific, US) and 0.5 μg/mL DAPI solution diluted in PBS was used to stain neutral lipids and nuclei, respectively. The intensities of the Bodipy signals were measured using Cytation 5 (Biotek), a fluorescence microplate reader.

### Long-term treatment of HepG2 OOCs with drugs

Following 24 h induction of steatosis with a mixture of OA and PA, HepG2 OOCs were treated daily by replacing the complete William’s E medium containing vehicle (serum-free medium or 0.1% DMSO), metformin (10, 100, and 1000 μM), pioglitazone (1 and 10 μM), or elafibranor (1, 10, and 50 μM) for 6 days.

### Albumin assay

The released amount of albumin in the conditioned medium was determined with a Human Albumin ELISA Kit (Abcam, US). The optical density at 450 nm was measured as an endpoint reading.

### Statistical analysis

We performed at least two independent experiments in which single or multiple OOC chamber(s) per treatment condition were used (as indicated by “n” in each figure legend). The data were analyzed with Prism 8 (GraphPad, US) and expressed as the mean ± SEM. Student’s t-test, one-way ANOVA, or two-way ANOVA with Tukey’s post hoc test was used for statistical analysis. The results were considered statistically significant if the probability value is less than 0.05.

### Supplementary Information

Supplementary Information.

Supplementary Legends.

Supplementary Figures.

## Data Availability

All data generated or analyzed during this study are included in this published article and its supplementary data files.

## Abbreviations

2D: Two dimensional
3D: Three dimensional
ACACA: Acetyl CoA carboxylase alpha
APOB: Apolipoprotein B
BSA: Bovine serum albumin
CPT1A: Carnitine palmitoyltransferase 1A
DGAT2: Diacylglycerol O-acetyltransferase 2
DMEM: Dulbecco’s modified eagle’s medium
DMSO: Dimethylsulfoxide
ECM: Extracellular matrix
FA: Fatty acid
FABP1: Fatty acid binding protein 1
FBS: Fetal bovine serum
FDA: Food and Drug Administration
LDH: Lactate dehydrogenase
MEM: Minimum essential media
NAFLD: Nonalcoholic fatty liver disease
NAS: NAFLD activity score
NASH: Nonalcoholic steatohepatitis
OA: Oleic acid
OOC: Organ-on-a-chip
PA: Palmitic acid
PNPLA2: Patatin like phospholipase domain containing 2
PBS: Phosphate-buffered saline
PPAR: Peroxisome proliferator-activated receptor
T2D: Type 2 diabetes
TAG: Triacylglycerol
