# Supplementary material for: A model of hepatic steatosis with declined viability and function in a liver-organ-on-a-chip
Source: Sci Rep. 2023 Oct 9;13:17019. doi: 10.1038/s41598-023-44198-0 (PMC10562420; doi:10.1038/s41598-023-44198-0)
Supplement: Supplementary file 1 — Supplementary Information. [file 41598_2023_44198_MOESM1_ESM.docx]

**Supplementary methods**

***RNA isolation, cDNA synthesis and real-time PCR.*** HepG2 cells were seeded in 6-well and OOC plates at initial cell densities of 0.3 x 10^6^ cells/well and 5 x 10^7^ cells/mL, respectively. On day 4, total RNA was extracted using TRIzol reagent (Invitrogen, US). The TRIzol-treated cell suspension of HepG2 OOCs was pooled from 16 OOC units for one sample. A total RNA miniprep kit (New England Biolabs, US) was used for DNase I treatment and RNA purification according to the manufacturer’s instruction. cDNAs were synthesized from 100 ng of total RNA using an iScript cDNA kit (Bio-Rad, US) and diluted 10 times with RNase-free water for the following step. TaqMan probes were purchased from Invitrogen (US) and listed in Table below. Levels of HepG2 transcripts were quantified by real-time PCR assay (Bio-Rad CFX96 Touch Real-Time PCR Detection System) using a Luna Universal Probe qPCR master mix (New England Biolabs, US) and calculated by the ΔCq method using GAPDH as a reference gene.

| Gene  symbol | TaqMan probe  assay ID |
| --- | --- |
|  |  |
| ACACA | Hs01046047_m1 |
| APOB | Hs00181142_m1 |
| CPT1A | Hs00912671_m1 |
| DGAT2 | Hs01045913_m1 |
| FABP1 | Hs00155026_m1 |
| PNPLA2 | Hs00982042_m1 |
| PPARA | Hs00947536_m1 |
| PPARD | Hs04187066_g1 |
| PPARG | Hs01115513_m1 |
| GAPDH | Hs99999905_m1 |

***Lipolysis assay.*** HepG2 OOCs (5 x 10^7^ cells/mL) were exposed to 0.5 mM OA for 24 h, followed by stimulating with vehicle (Veh, 0.1% DMSO) or 10 μM isoproterenol (Isop, a beta-agonist) that can induce lipolysis in hepatocytes^1^ for 6h. The released amount of glycerol, a product of lipolysis, in the conditioned medium was determined with a glycerol kit (Abcam, US).

***Induction of steatosis in HepG2 OOCs with high concentrations of glucose and insulin.*** At 24 h after seeding, HepG2 OOCs were exposed to serum-free Minimum Essential Medium (MEM, Gibco, US) containing a low concentration of glucose (LG, 5.5 mM) or a combination of high concentrations of glucose (HG, 30 mM) and insulin (Ins, 100 nM) for 24 h.

1 Schott, M. B. *et al.* beta-Adrenergic induction of lipolysis in hepatocytes is inhibited by ethanol exposure. *J Biol Chem* **292**, 11815-11828, doi:10.1074/jbc.M117.777748 (2017).
