# Supplementary material for: A model of hepatic steatosis with declined viability and function in a liver-organ-on-a-chip
Source: Sci Rep. 2023 Oct 9;13:17019. doi: 10.1038/s41598-023-44198-0 (PMC10562420; doi:10.1038/s41598-023-44198-0)
Supplement: Supplementary file 2 — Supplementary Legends. [file 41598_2023_44198_MOESM2_ESM.docx]

**Supplementary figure 1. Comparison of 2D and 3D platforms for HepG2 cultures.**

HepG2 cells were grown in 96-well plates (2D) or OrganoPlate 2-lane plates with Matrigel (3D) for 14 days. (A) Albumin production in conditioned medium, (B) LDH activity, and (C) A representative image of HepG2 OOC cells grown in Matrigel ECM (bottom) and medium path (top) on Day 14. Data are presented as mean ± SEM and analyzed by a two-way repeated measures ANOVA (n=5-10). * p<0.05 vs. each day of 2D.

**Supplementary figure 2. mRNA expression levels of key regulators involved in lipid metabolism in 2D and OOC cultures of HepG2.**

HepG2 cells were cultured in 6-well (2D) or OOC plates for 4 days. Data are presented as mean ± SEM and analyzed by Student’s t-test (n=5). * p<0.05 vs. 2D.

**Supplementary figure 3. Induction of de novo lipogenesis (A) and lipolysis (B) in HepG2 OOCs.**

(A) HepG2 OOC cells were treated with a low concentration of glucose (LG, 5.5 mM) or high concentrations of glucose (HG, 30 mM) and insulin (Ins, 100 nM) for 24 h. Following staining with Bodipy dye, the fluorescence intensity was quantified by Cytation 5. (B) HepG2 OOC cells were treated with 0.5 mM oleic acid (OA) for 24 h, followed by stimulating with vehicle (Veh, 0.1% DMSO) or 10 μM isoproterenol (Isop, a beta-agonist). Released amounts of glycerol into conditioned media were measured by a lipolysis assay kit. Data are presented as mean ± SEM and analyzed by Student’s t-test (n=3-4). * p<0.05 vs. Veh.

**Supplementary figure 4. Induction of steatosis in HepG2 2D cultures by exposure to palmitic acid.**

Quantitative analysis of Bodipy fluorescence in HepG2 2D cultures treated with vehicle (Veh, 1 mM NaOH) or palmitic acid (PA, 0.5 mM) for 24 h. Data are presented as mean ± SEM and analyzed by Student’s t-test (n=6). * p<0.05 vs. Veh.
