# Supplementary figures and images for: A model of hepatic steatosis with declined viability and function in a liver-organ-on-a-chip
Source: Sci Rep. 2023 Oct 9;13:17019. doi: 10.1038/s41598-023-44198-0 (PMC10562420; doi:10.1038/s41598-023-44198-0)

**Supplementary Figure 1.**

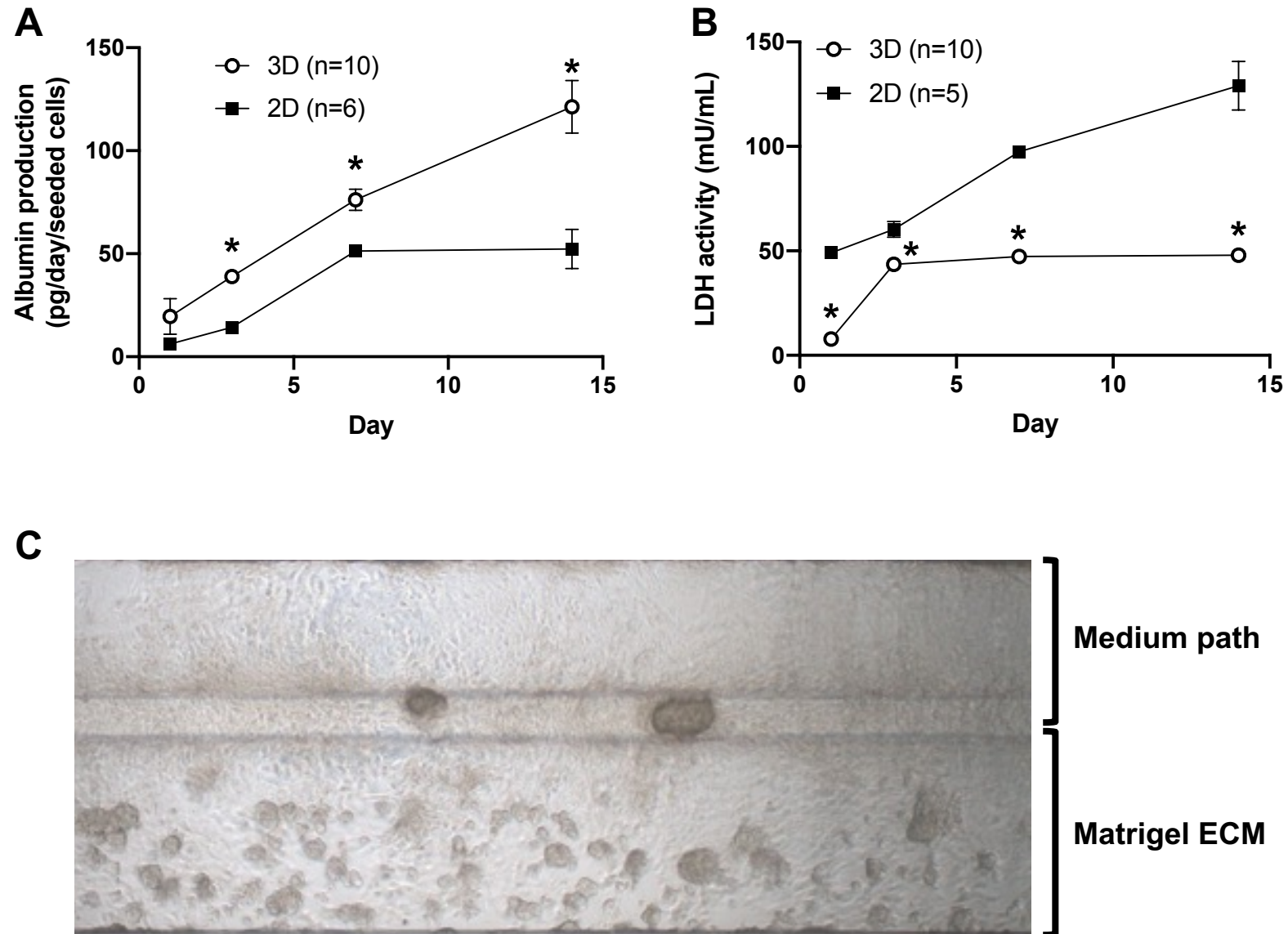

Supplementary Figure 2.

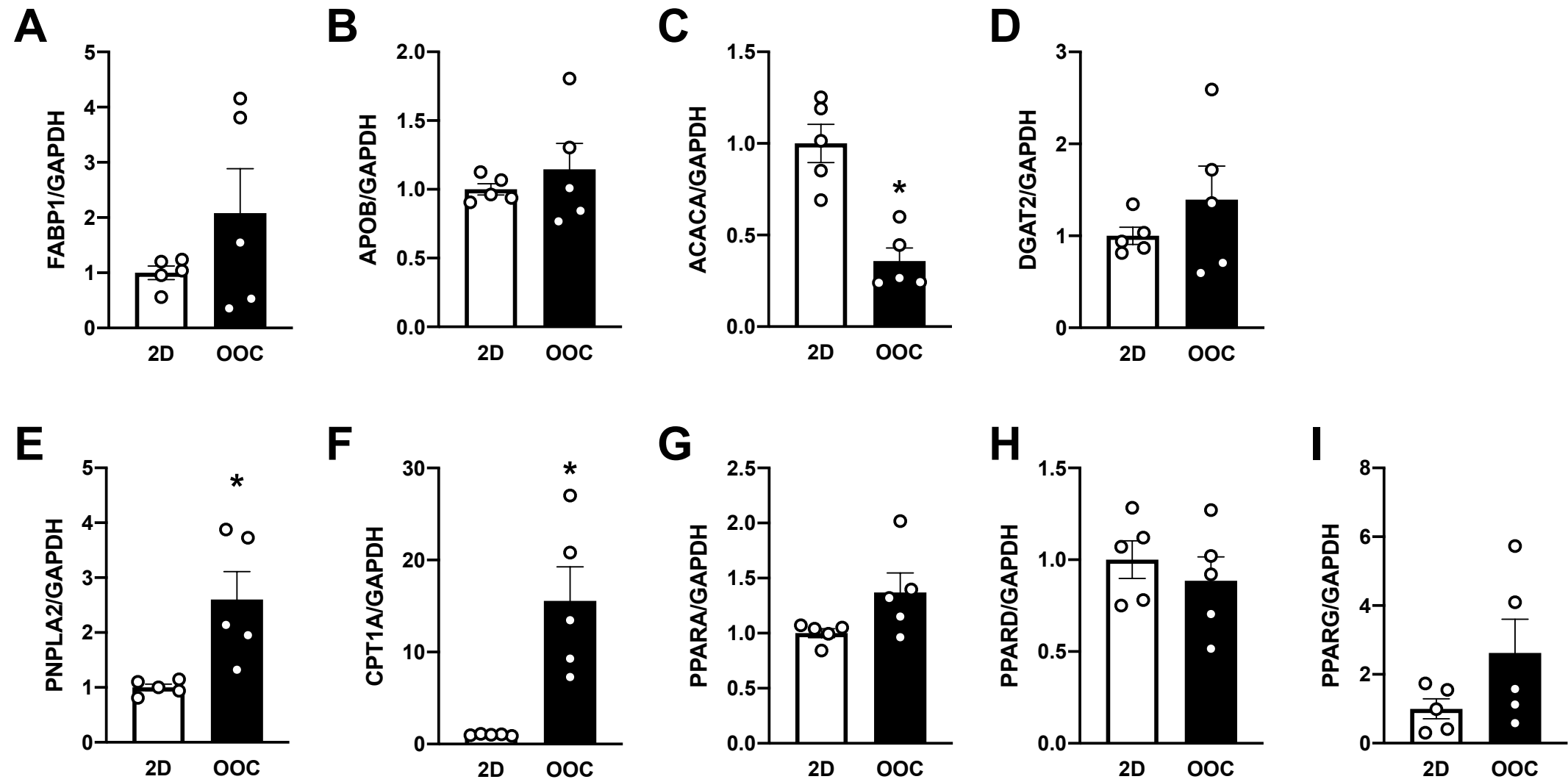

Supplementary Figure 3.

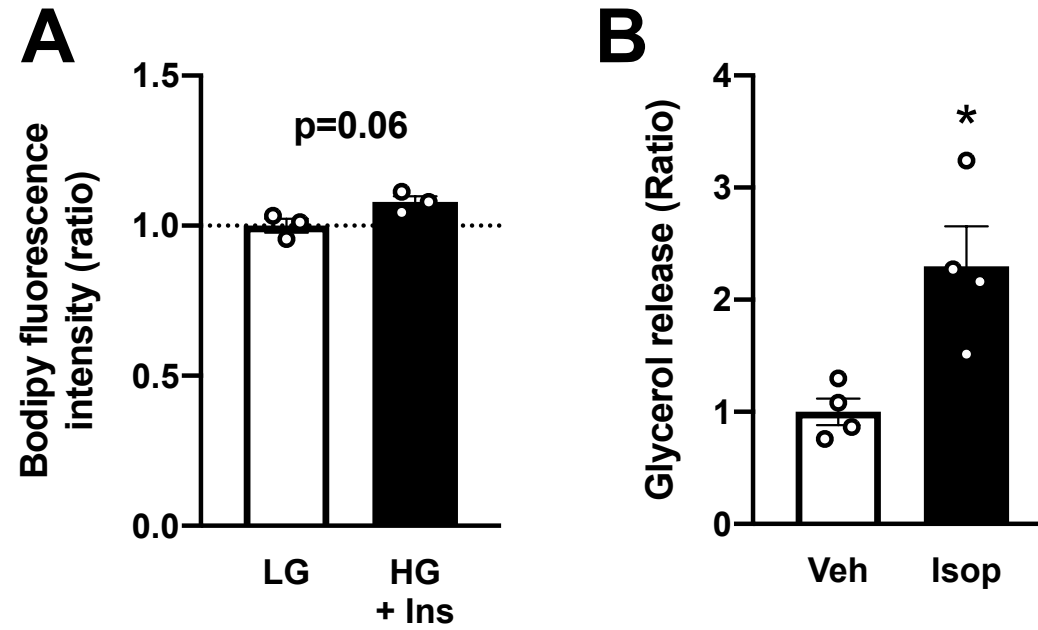

Supplementary Figure 4.

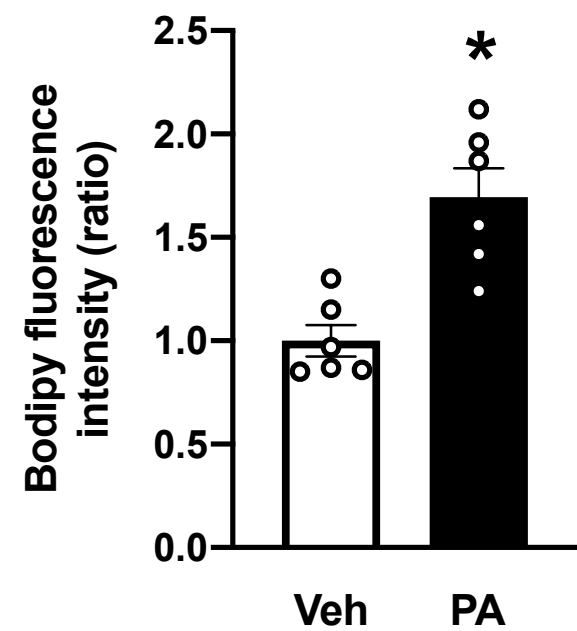

Supplement: Supplementary file 3 — Supplementary Figures. [file 41598_2023_44198_MOESM3_ESM.pdf]
